# Metabolic Engineering Plant Seeds with Fish Oil-Like Levels of DHA

**DOI:** 10.1371/journal.pone.0049165

**Published:** 2012-11-07

**Authors:** James R. Petrie, Pushkar Shrestha, Xue-Rong Zhou, Maged P. Mansour, Qing Liu, Srinivas Belide, Peter D. Nichols, Surinder P. Singh

**Affiliations:** 1 CSIRO Food Futures National Research Flagship, Canberra, Australian Capital Territory, Australia; 2 CSIRO Plant Industry, Canberra, Australian Capital Territory, Australia; 3 CSIRO Marine and Atmospheric Research, Hobart, Tasmania, Australia; Ghent University, Belgium

## Abstract

**Background:**

Omega-3 long-chain (≥C_20_) polyunsaturated fatty acids (ω3 LC-PUFA) have critical roles in human health and development with studies indicating that deficiencies in these fatty acids can increase the risk or severity of cardiovascular and inflammatory diseases in particular. These fatty acids are predominantly sourced from fish and algal oils, but it is widely recognised that there is an urgent need for an alternative and sustainable source of EPA and DHA. Since the earliest demonstrations of ω3 LC-PUFA engineering there has been good progress in engineering the C_20_ EPA with seed fatty acid levels similar to that observed in bulk fish oil (∼18%), although undesirable ω6 PUFA levels have also remained high.

**Methodology/Principal Findings:**

The transgenic seed production of the particularly important C_22_ DHA has been problematic with many attempts resulting in the accumulation of EPA/DPA, but only a few percent of DHA. This study describes the production of up to 15% of the C_22_ fatty acid DHA in *Arabidopsis thaliana* seed oil with a high ω3/ω6 ratio. This was achieved using a transgenic pathway to increase the C_18_ ALA which was then converted to DHA by a microalgal Δ6-desaturase pathway.

**Conclusions/Significance:**

The amount of DHA described in this study exceeds the 12% level at which DHA is generally found in bulk fish oil. This is a breakthrough in the development of sustainable alternative sources of DHA as this technology should be applicable in oilseed crops. One hectare of a *Brassica napus* crop containing 12% DHA in seed oil would produce as much DHA as approximately 10,000 fish.

## Introduction

**Figure 1:**
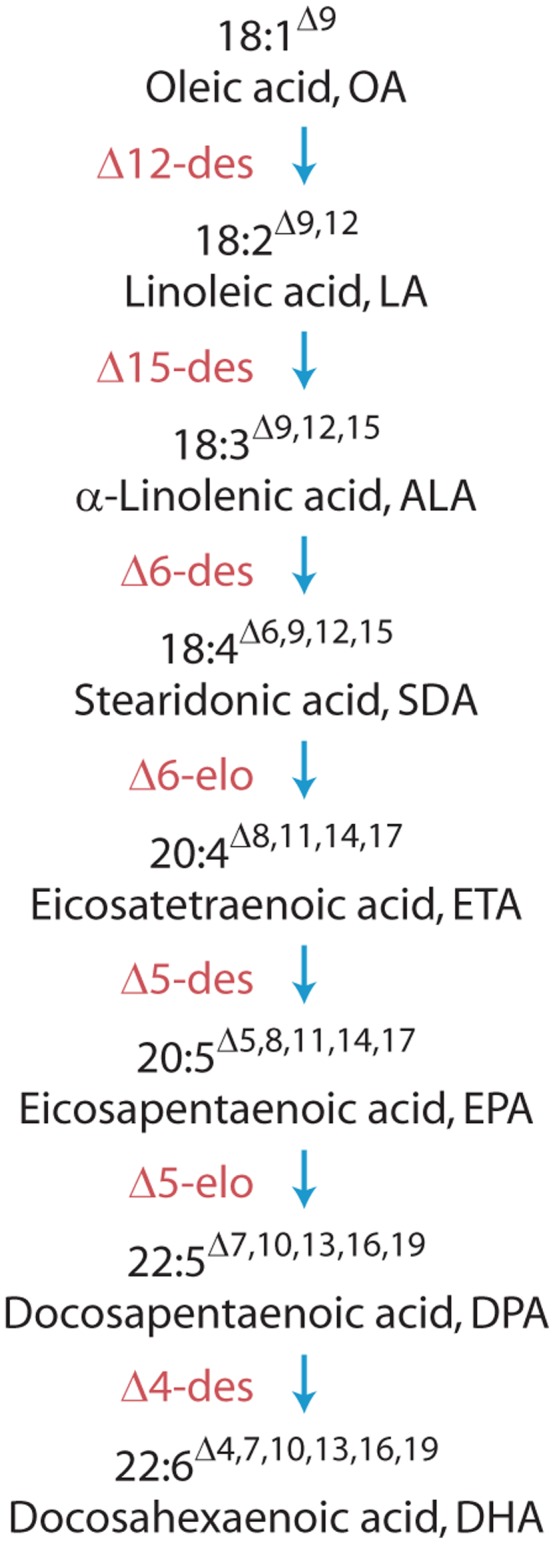
DHA biosynthesis pathway described in this study where ‘des’ refers to desaturase and ‘elo’ to elongase.

Metabolic engineering of omega-3 long-chain (≥C_20_) polyunsaturated fatty acids (ω3 LC-PUFA, **Figure 1**) has been a key metabolic engineering target in recent years. The two main ω3 LC-PUFA are eicosapentaenoic acid (EPA, 20∶5ω3) and docosahexaenoic acid (DHA, 22∶6ω3). The dietary intake of preformed ω3 LC-PUFA is important [1] since *in vivo* conversion of C_18_ fatty acids to DHA is relatively poor [2]. This is especially relevant for brain development in infants [3] and for aspects of cardiovascular health [4]. These factors have resulted in the inclusion of DHA in infant formulae now being widespread and pharmaceutical-grade ω3 LC-PUFA therapies are expanding rapidly for treatment of certain cardiovascular-related diseases. Demand for nutraceutical ω3 LC-PUFA products, including DHA-specific products, is growing rapidly and an additional, sustainable source of ω3 LC-PUFA is required to complement the existing marine fish oil supply [5].

Good progress has been made in engineering the C_20_ EPA with groups reporting the seed production of levels similar to that observed in bulk fish oil (∼18%), although ω6 LC-PUFA levels also remained high in these examples [6]–[7]. The conversion of this C_20_ fatty acid to the particularly important C_22_ DHA, however, has been problematic with many attempts resulting in the accumulation of EPA/DPA and little DHA [6]–[9]. The difficulties associated with achieving high levels of DHA accumulation have been described [10]–[11] with key challenges including the reduction of undesirable ω6 fatty acid co-production, achieving a continuous flux of substrates throughout the entire pathway without large losses to metabolically inactive pools and improvement of the critical Δ5-elongase efficiency to convert EPA to DPA (**Figure 1**). We here report the construction and characterisation of an engineered pathway that largely overcomes these challenges, resulting in the accumulation of up to 15% DHA in the seed oil of *Arabidopsis thaliana*.

## Results and Discussion

**Figure 2:**
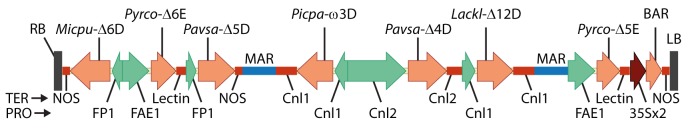
Construct map of pJP3416_GA7 left-right border (LB and RB) region. Abbreviations are: TER, terminator or polyadenylation region; PRO, promoter; NOS, *Agrobacterium tumefaciens* nopaline synthase terminator; FP1, *Brassica napus* truncated napin promoter; FAE1, *Arabidopsis thaliana* FAE1 promoter; Lectin, *Glycine max* lectin terminator; Cnl1 and Cnl2 denotes the *Linum usitatissimum* conlinin1 or conlinin2 promoter or terminator. MAR denotes the Rb7 matrix attachment region from *Nicotiana tabacum* and gene identities are given in the text and the Materials and Methods section.

The insert region of the binary vector pJP3416_GA7 (**Figure 2**) contained seven fatty acid biosynthesis genes driven by seed-specific promoters and a constitutively-expressed plant selectable marker. The transgenic pathway was designed to convert oleic acid (OA) to DHA (**Figure 1**) and consisted of the *Lachancea kluyveri* Δ12-desaturase, *Pichia pastoris* Δ15−/ω3-desaturase, *Micromonas pusilla* Δ6-desaturase, *Pyramimonas cordata* Δ6- and Δ5-elongases and *Pavlova salina* Δ5- and Δ4-desaturases. This combination of microalgal genes, with the exception of the yeast Δ12- and Δ15−/ω3-desaturases, had previously resulted in an efficient conversion of native plant substrate fatty acids to DHA (up to 15.9% in leaf TAG) in the transient *Nicotiana benthamiana* assay system [11]. In pJP3416_GA7, these genes were expressed by the *Brassicaceae*-active seed-specific promoters *A. thaliana* FAE1, *Linum usitatissimum* conlinin1 (Cnl1) and conlinin2 (Cnl2) and the truncated *Brassica napus* napin promoter (FP1) with the tobacco mosaic virus 5′ untranslated enhancer leader sequence upstream of each fatty acid biosynthesis gene.

**Figure 3:**
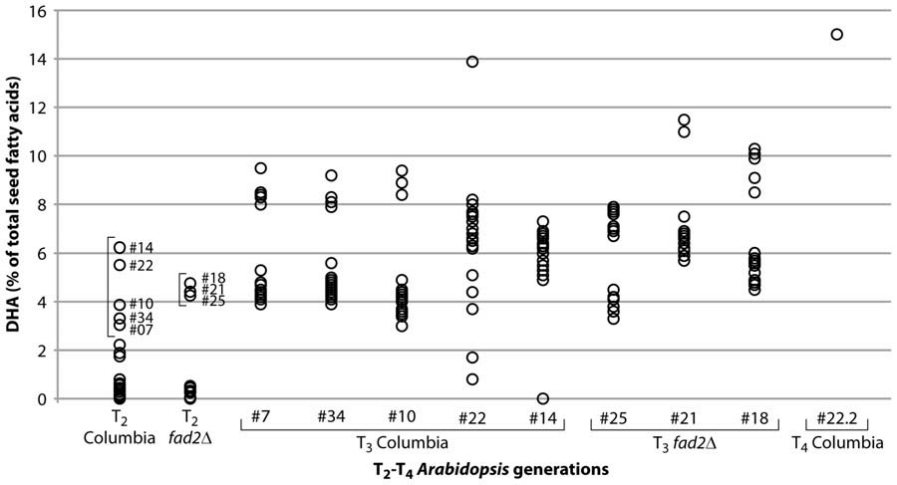
DHA levels as a percentage of total seed fatty acids from multiple independent transgenic *Arabidopsis thaliana* events in the T_2_ and T_3_ generations and the best T_4_ event. The bracketed T_2_ events were taken to T_3_. Events from both the Columbia and *fad2* mutant *A. thaliana* backgrounds are shown.

The construct was transformed in the *A. thaliana* ecotype Columbia and a *fad2* mutant (see **Table 1** for seed fatty acid profiles of parentals). T_1_ seeds from dipped plants were selected for phosphinothricin (PPT) resistance and T_2_ seed from surviving plants was harvested and analysed for fatty acid composition (**Figure 3** **,** **Table 1**). Several lines, indicated by brackets in **Figure 3**, were again selected for PPT resistance and resistant seedlings for each line transferred to soil. T_3_ seeds from these plants were harvested and their seed oil-derived fatty acids were analysed by GC (**Table 1**) which revealed that pJP3416_GA7 was functioning to generate significant levels of ω3 LC-PUFA in seed oil. Up to 13.9% DHA was observed in the best T_3_ event (Columbia#22) with a total of 24.3% new ω3 fatty acids. Similarly, the best event in the *fad2* mutant background yielded 20.6% total new ω3 fatty acids including 11.5% DHA (**Table 1**). In contrast, new transgenic ω6 fatty acids were found in very low relative levels (**Table 1**).

**Table 1:** Representative fatty acid profiles of total pooled seed lipids from independent transgenic parental, T_2_, T_3_ and T_4_ *Arabidopsis* lines with the highest DHA levels.

		*fad2* mutant	T_2_ FAD2_21	T_3_ FAD2-21.2	Columbia	T_2_ Col_22	T_3_ Col_22.2	T_4_ Col_22.2 (mean±SD)	T_4_ Col_22.2 best line
	16∶0	6.6	8.5	10.7	8.1	8.3	11.2	10.6±0.9	12.2
	18∶0	3.1	3.9	3.8	3.7	3.4	3.4	3.5±0.4	3.6
	18∶1ω7	1.6	2.0	2.2	1.7	2.3	2.9	2.3±0.2	2.6
	20∶0	1.6	2.2	2.0	1.9	1.6	1.4	1.9±0.3	2.0
	20∶1ω7	1.6	1.7	1.4	1.8	1.5	1.6	1.6±0.2	1.9
	20∶1ω9/11	19.7	15.9	12.4	18.0	13.9	9.5	11.7±1.7	9.5
	22∶1ω9	1.3	1.3	1.1	1.5	1.0	0.6	0.9±0.1	0.8
	Minor	1.6	1.9	2.1	1.7	2.2	2.3	2.3±0.1	2.6
	OA	37.0	8.2	4.2	12.5	10.0	4.6	4.6±1.0	3.3
	LA	11.9	16.6	8.9	29.1	13.7	5.6	5.3±0.9	4.3
	ALA	13.3	27.7	28.9	18.3	30.4	31.5	31.0±1.1	29.5
**Omega-6**	GLA		0.2	0.6		0.2	0.4	0.4±0.1	0.4
	20∶2ω6	0.7	1.4	1.2	1.7	1.0	0.7	0.9±0.1	0.9
	DGLA								
	ARA								
	22∶4ω6								
	DPA6								
**Omega-3**	SDA		1.8	5.2		2.7	5.3	4.8±0.9	5.5
	20∶3ω3		0.8	1.3		0.6	1.3	1.5±0.2	1.7
	ETA		0.8	0.9		0.8	0.9	0.8±0.2	0.8
	EPA		0.4	1.0		0.7	1.9	1.5±0.3	1.8
	DPA		0.3	0.6		0.2	1.0	1.1±0.2	1.5
	**DHA**		**4.4**	**11.5**		**5.5**	**13.9**	**13.3±1.6**	**15.1**
	**Δ12-des**	55%	90%	95%	84%	88%	94%	79%	83%
	**Δ15-des**	51%	66%	82%	37%	73%	89%	89%	91%
**Omega-3**	**Δ6-des**		21%	39%		24%	41%	41%	46%
	**Δ6-elo**		77%	73%		73%	77%	78%	78%
	**Δ5-des**		86%	93%		89%	95%	95%	96%
	**Δ5-elo**		92%	92%		90%	89%	91%	90%
	**Δ4-des**		93%	95%		96%	93%	92%	91%

‘Col’ refers to the Columbia ecotype and ‘FAD2’ to the *fad2* mutant. The standard error shown in the T_4_ generation denotes the SD of n = 10. Apparent conversion efficiencies shown at the bottom describe the ω3 pathway and are calculated as the sum of product FAs/sum of substrate + product FAs.

**Figure 4:**
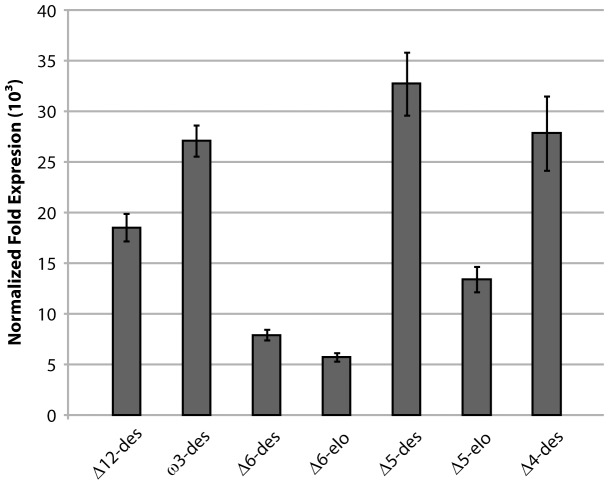
Real-Time quantitative PCR of transgenes in developing DHA seed. Normal fold expression was calculated using the ΔΔCt method using a fatty acid biosynthesis gene, β-ketoacyl-acyl carrier protein synthase II (KASII), as the reference gene.

Seeds from the Columbia#22 line were planted directly to soil. Southern blot analysis of pooled material from this generation found that this line was triple-copy for the pJP3416_GA7 construct (data not shown). Seeds from mature plants were harvested and analysed. Some variation in DHA composition was observed in this generation (13.3%±1.6, **Table 1**), suggesting that this three-copy event was not yet homozygous, although no variation in germination rate or seedling establishment was observed. This was further indicated by the DHA level in the best line being further increased to 15.1%, with the fatty acid profiles largely similar to the T_3_ generation (**Table 1**). Real-Time quantitative PCR was performed on cDNA generated from total RNA isolated from developing T_4_ embryos using a fatty acid biosynthesis gene, β-ketoacyl-acyl carrier protein synthase II (KASII), as the reference gene **(** **Figure 4** **)**. The Δ6-desaturase and Δ6-elongase were found to be expressed relatively poorly compared to the other transgenes.

**Figure 5:**
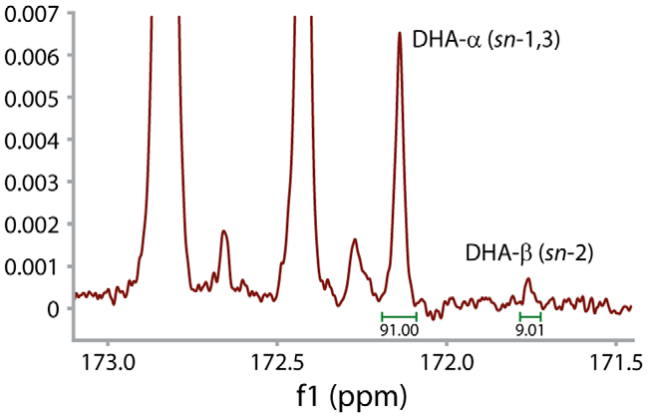
NMR trace showing the positional distribution of DHA on the *A. thaliana* TAG with the *sn*-1 and *sn*-3 positions indicated by DHA-α and *sn*-2 by DHA-β. DHA is preferentially positioned at *sn*-1/3.

**Figure 6:**
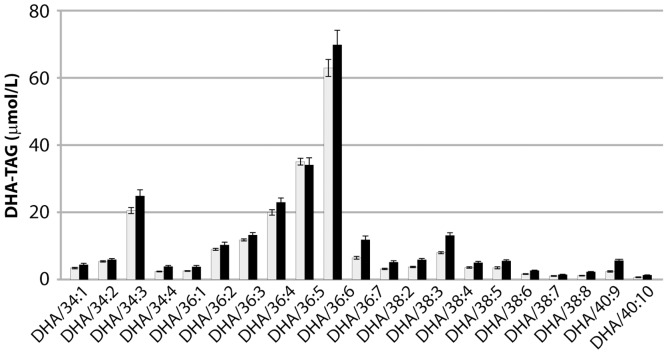
LC-MS analysis of major DHA-containing triacylglycerol species in transgenic *Arabidopsis thaliana* developing (grey) and mature (black) seeds. The number following the DHA denotes the total number of carbon atoms and total number of double bonds in the other two fatty acids.

^13^C NMR regiospecificity analysis was performed on DHA-containing *A. thaliana* seed oil to determine the positional of the DHA fatty acid on the TAG. We found that the *sn*-1/3 position of TAG was significantly enriched for DHA (**Figure 5**) with little DHA observed at the *sn*-2 position. This is especially interesting since the enrichment at the *sn*-2 position was recently observed for engineered arachidonic acid (a C_20_ ω6 LC-PUFA) in *Brassica napus* [14]. It will be important to observe the positional distribution of DHA in other engineered species. Finally, the total lipid was also analysed by triple quadrupole LC-MS to determine the major DHA-containing triacylglycerol (TAG) species (**Figure 6**). The most abundant DHA-containing TAG species was found to be DHA-18∶3-18∶3 (nomenclature not descriptive of positional distribution) with the second-most abundant being DHA-18∶3-18∶2. Tri-DHA TAG was observed in total seed oil, albeit at low quantities. The two major DHA-containing TAG were further confirmed by Q-TOF MS/MS (data not shown).

pJP3416_GA7 was designed to meet multiple functional objectives. First, we focused on producing DHA to the exclusion of intermediate and ω6 fatty acids. To achieve this, adequate seed expression of all transgenes was required. The promoters used in this construct had either been previously described or tested in our laboratory for strong seed-specific function in *A. thaliana*. Second, intergenerational stability without gene silencing was required and we thus avoided a simple linear design [9] with identical promoters and polyadenylation regions for each expression cassette. Instead, Rb7 matrix attachment regions from *Nicotiana tabacum* were used as spacer DNA to separate the three-cassette inverted segments which had previously been found to operate effectively (data not shown). Third, correct expression timing was important so that the promoters expressing the first part of the pathway would not be active before the promoters expressing subsequent genes in the biosynthetic pathway. For example, we had found the FAE1 promoter tended to be active early in seed development relative to FP1, Cnl1 and Cnl2 and therefore did not use this promoter to express a gene where a native plant substrate was available. This was intended to avoid the accumulation of intermediates since transgenic fatty acids were only produced when the subsequent enzyme in the pathway was already expressed.

The use of the *A. thaliana* ecotype Columbia and a *fad2* mutant provided a good contrast between DHA production in seed naturally high in the polyunsaturated LA and ALA as well as seed which contained little of these fatty acids but was rich in the precursor OA. One of the key challenges of LC-PUFA engineering in plant seeds is the loss of pathway intermediates from metabolically active acyl-PC and acyl-CoA pools to TAG before they can be further elongated or desaturated [10]. Earlier studies had demonstrated that ALA in particular was susceptible to accumulation in TAG and we decided to test whether the transgenic production of LA and ALA in the *fad2* mutant would result in greater Δ6-desaturase accessibility. However, pJP3416_GA7 resulted in good DHA production in both backgrounds (**Table 1**) and whilst the highest DHA line described above was found in the Columbia background, the averages of the selected T_3_ populations were roughly equal with 9.7% DHA in the Columbia background and 9.9% in the *fad2* mutant. This is likely due to the high activity of the *L. kluyveri* Δ12-desaturase which actually yielded slightly higher Δ12-desaturation in the best *fad2* mutant event than the best Columbia event (**Table 1**). This indicates that native FAD2 activity may not the most important factor in crop selection although it is important to note that the ratio between OA and polyunsaturates can be indicative of other important biochemical differences in a seed.

There were several notable characteristics of this pathway. For instance, the ratio of ω3/ω6 fatty acids was 8/1 when the native substrates LA and ALA were included or 16/1 if these were excluded. This was likely due both to the ω3 preference of the *M. pusilla* Δ6-desaturase [12] and the presence of the broad-specificity *P. pastoris* ω3-desaturase. This is an extremely desirable quality for an ω3 oil due to the opposing, pro-inflammatory effects of ω6 fatty acids [1]. It was also worth noting the low level of intermediate fatty acids in the seed oil. Previous attempts to produce DHA have resulted in the accumulation of high levels of EPA but relatively little conversion to DPA and DHA. Independent pJP3416_GA7 events had consistently high Δ5-elongation and subsequent Δ4-desaturation indicating that the EPA to DPA conversion hurdle had been overcome in these lines. The Δ6-desaturase had consistently low activity relative to the other transgenes. The *M. pusilla* enzyme is likely acyl-CoA in nature and performs well alongside comparable enzymes in yeast and other assay systems [12], but has never exceeded approximately 50% ALA conversion in plant systems. Similar results have been observed by other groups and it has been proposed that ALA conversion by acyl-CoA desaturases can be limited in certain plant species [13]. The Δ6-desaturase was also expressed relatively poorly and it is likely that this contributed to the relatively low activity.

The pJP3416_GA7 vector had several limitations which require further work to fully understand and overcome. Real-Time PCR showed that the expression levels of individual transgenes varied considerably with the Δ6-desaturase and Δ6-elongase having the lowest relative expressions. These genes also had the lowest apparent conversion efficiencies resulting in the accumulation of significant ALA and SDA and this is a likely cause of the gene dosage effect that resulted in a triple-copy event yielding the highest DHA accumulation. Future work will focus on improving construct design to result in stronger gene expression to reduce the gene dosage effect. It will also be important to examine the lipidome of developing transgenic DHA seeds to better understand how the pathway operates in the seed lipid pools. Given the high efficiency of the pathway in converting ETA through to DHA, it is tempting to speculate that the *P. salina* Δ5- and Δ4-desaturases are able to utilise acyl-CoA substrates; further work is required to confirm this. Finally, the impact of underlying plant seed biochemistry on DHA production can be explored by transforming this pathway in other species.

In conclusion, the production of high levels of DHA has been a major goal of the metabolic engineering community. This study resulted in the accumulation of up to 15% DHA in a land plant seed oil, a level that exceeds the 12% level generally found in commodity bulk fish oil. A high ω3/ω6 ratio was also observed. We look forward to the application of this technology in crop species: 1 hectare of a *Brassica napus* crop containing 12% DHA in seed oil would produce as much DHA as approximately 10,000 fish. (This is a simplified calculation based on 10,000 kg fish = 1,000 kg oil = 120 kg DHA. Assumptions are that average fish = 1 kg, fish oil yield is 10% by mass, average DHA is 12%. For smaller size and less oily fish, the number of equivalent fish increases and for larger fish, the number of fish would decrease. Similarly, 1 Ha *B. napus* = 2.5 T seed = 1,000 kg oil = 120 kg DHA. Assumptions are that *B. napus* seed contains 40% oil by weight, 12% DHA.).

## Materials and Methods

### Binary Vector Construction and *A. thaliana* Transformation

Fatty acid biosynthesis gene sequences were sourced from yeast and microalgae. The *L. kluyveri* Δ12-desaturase (Genbank accession BAD08375) was identified by BLAST using known Δ12-desaturase sequences as queries, whilst the other yeast gene, the *P. pastoris* Δ15−/ω3-desaturase, had been characterised as having a broad ω3-specificity [15]. The microalgal genes had also been previously described and tested in the transient *N. benthamiana* leaf assay system under the control of both constitutive and seed specific promoters as well as stable seed expression [9] [11]–[12]. The seed-specific promoters used in this study had all been previously described: *A. thaliana* FAE1 [16], *L. usitatissimum* Cnl1 and Cnl2 [17]–[18] and the truncated *B. napus* napin promoter [19]. The vector was constructed by synthesising (Geneart, Regensburg, Germany) the seven fatty acid biosynthesis expression cassettes with MAR spacers [20]–[21] and tobacco mosaic virus 5′ untranslated enhancer leader sequences [22] as a single 19.75 kb fragment flanked by *Not*I restriction sites. The fragment was then cloned into a binary vector, pJP3416, at a *Psp*OMI site. pJP3416 contained the constitutively-expressed *Streptomyces viridochromogenes* phosphinothricin-N-acetyltransferase gene to confer phosphinothricin (PPT) resistance. *A. thaliana* ecotype Columbia and a *fad2* mutant [23] were transformed by *Agrobacterium*-mediated floral dip [24] and seeds selected for PPT resistance by germination and establishment on MS media plates containing 3.5 mg/L PPT. DNA was extracted and Southern blots performed according to established protocols [25]. Total RNA was extracted using an RNeasy mini-kit (QIAGEN, Doncaster, VIC, Australia) and RT-PCR performed using a OneStep RT-PCR Kit (QIAGEN).

### Fatty Acid Profile Analysis

Fatty acid profiles were determined on batches of approximately 200 seeds. Fatty acid methyl esters (FAME) were prepared by incubating seeds in 1N methanolic HCl at 80°C for 2 hours in a glass tube fitted with Teflon-lined screw cap and FAME extracted in hexane before analysis by gas chromatography (GC) using an Agilent Technologies 7890A GC (Palo Alto, California, USA) essentially as described by [26], but equipped with a 30 m SGE-BPX70 column. Peaks were quantified with Agilent Technologies ChemStation software (Rev B.04.03 (16), Palo Alto, California, USA) based on the response of the known amount of the external standard GLC-411 (Nucheck Prep, Elysian, MN, USA). Selected samples were also analysed on a non-polar GC column together with a GC-MS system for further confirmation of FA identification and component quantification. GC was performed using an Agilent Technologies 6890N GC (Palo Alto, California, USA) equipped with a non-polar Equity™-1 fused silica capillary column (15 m×0.1 mm i.d., 0.1 µm film thickness), an FID, a split/splitless injector and an Agilent Technologies 7683 Series auto sampler. Helium was the carrier gas. Samples were injected in splitless mode at an oven temperature of 120°C. After injection, oven temperature was raised to 270°C at 10°C min^−1^ and finally to 300°C at 5°C min^−1^. Peaks were quantified with Agilent Technologies ChemStation software. Individual component identifications were confirmed by mass spectral data and by comparing retention time data with authentic and laboratory standards. GC-mass spectrometric (GC-MS) analyses were performed on a Finnigan Thermoquest GCQ GC-MS fitted with an on-column injector and using Thermoquest Xcalibur software (Austin, Texas, USA). The GC was equipped with an HP-5 cross-linked methyl silicone fused silica capillary column (50 m×0.32 mm i.d.) of similar polarity to that described above. Helium was used as carrier gas, with operating conditions previously described [27].

### Lipid Extraction and ^13^C NMR Regiospecificity Analysis

Total lipid was extracted by first crushing seeds under hexane before transferring to a glass tube containing 10 mL hexane. The tube was warmed at approximately 55°C in a water bath, vortexed and centrifuged. The hexane solution was removed and the procedure repeated with a further 4×10 mL. The extracts were combined, concentrated by rotary evaporation and the TAG purified by eluting through a short silica column using 20 mL of 7% diethyl ether in hexane. Acyl group positional distributions on TAG were determined as previously described [12] using tuna oil as a *sn*-2 DHA comparator.

### Real-Time Quantitative PCR

Gene expression analysis was performed using a BioRad CFX96™ Real-Time PCR (BioRad, Hercules, CA, USA). Gene-specific primers were designed to have similar melting temperatures and amplify ∼200 bp fragments. PCR reactions were performed in triplicate in 10 µL volumes consisting of the iQTM SYBR Green supermix (BioRad), 5 µM each primer and 400 ng cDNA. The cycling conditions were 1×95°C for 3 min., 40×95°C for 10 sec., 60°C for 30 sec., 68°C for 30 sec. The endogenous fatty acid biosynthesis gene β-ketoacyl-acyl carrier protein synthase II (KASII) was used as a reference with data calibrated relative to each gene expression level following the 2^−ΔΔCt^ method.

### TAG Species Analysis with LC-MS

Total lipids were extracted from freeze-dried developing (twelve days after flowering) and mature seeds with tri-C17∶0-TAG as internal standard. The extracted lipids were dissolved into 1 mL of 10 mM butylated hydroxytoluene in butanol:methanol (1∶1, vol.) per 5 mg dry materials and analysed using an Agilent 1200 series LC and 6410B electrospray ionisation triple quadrupole LC-MS. Lipids were chromatographically separated using an Ascentis Express RP-Amide column (50 mm×2.1 mm, 2.7 µm, Supelco) and a binary gradient with a flow rate of 0.2 mL/min. The mobile phases were: A. 10 mM ammonium formate in H_2_O:methanol: tetrahydrofuran (50∶20:30, vol.); B. 10 mM ammonium formate in H_2_O:methanol: tetrahydrofuran (5∶20:75, vol.). Multiple reaction monitoring (MRM) lists were based on the following major fatty acids: 16∶0, 18∶0, 18∶1, 18∶2, 18∶3, 18∶4, 20∶1, 20∶2, 20∶3, 20∶4, 20∶5, 22∶4, 22∶5, 22∶6 using a collision energy of 30 V and fragmentor energy of 60 V. Individual MRM TAG was identified based on ammoniated precursor ion and product ion from neutral loss of 22∶6. TAG was quantified using the 10 µM tristearin external standard.
